# Vibrational Spectroscopy-Based Chemometrics Analysis of *Clinacanthus nutans* Extracts after Postharvest Processing and Extract Effects on Cardiac C-Kit Cells

**DOI:** 10.1155/2022/1967593

**Published:** 2022-02-23

**Authors:** Vuanghao Lim, Hui Wen Chong, Nozlena Abdul Samad, Siti Aisyah Abd Ghafar, Ida Shazrina Ismail, Rafeezul Mohamed, Yoke Keong Yong, Chee Yuen Gan, Jun Jie Tan

**Affiliations:** ^1^Advanced Medical and Dental Institute, Universiti Sains Malaysia, Bertam 13200, Kepala Batas, Penang, Malaysia; ^2^Department of Oral Biology and Basic Sciences, Faculty of Dentistry, Universiti Sains Islam Malaysia, Persiaran MPAJ, Jalan Pandan Indah 55100 Pandan Indah, Ampang, Malaysia; ^3^Department of Human Anatomy, Faculty of Medicine and Health Sciences, Universiti Putra Malaysia, 43400 Serdang, Selangor, Malaysia; ^4^Analytical Biochemistry Research Centre, University Innovation Incubator Building, SAINS@USM Campus, 11900 Bayan Lepas, Penang, Malaysia

## Abstract

Chemical constituents in plants can be greatly affected by postharvest processing, and it is important to identify the factors that lead to significant changes in chemistry and bioactivity. In this study, attenuated total reflectance-Fourier transform infrared (ATR-FTIR) spectroscopy was used to analyze extracts of *Clinacanthus nutan (C. nutans)* leaves generated using different parameters (solvent polarities, solid-liquid ratios, ultrasonic durations, and cycles of extraction). In addition, the effects of these extracts on the viability of cardiac c-kit cells (CCs) were tested. The IR spectra were processed using SIMCA-P software. PCA results of all tested parameter sets were within acceptable values. Solvent polarity was identified as the most influential factor to observe the differences in chemical profile and activities of *C. nutans* extracts. Ideal extraction conditions were identified, for two sample groups (G1 and G2), as they showed optimal total phenolic content (TPC) yield of 44.66 ± 0.83 mg GAE/g dw and 45.99 ± 0.29 mg GAE/g dw and CC viability of 171.81 ± 4.06% and 147.53 ± 6.80%, respectively. Validation tools such as CV-ANOVA (*p* < 0.05) and permutation (*R*^2^ and *Q*^2^ plots were well intercepted to each other) have further affirmed the significance and reliability of the partial least square (PLS) model of solvent polarity employed in extraction. Hence, these approaches help optimize postharvest processes that encourage positive TPC and CCs results in *C. nutans* extracts.

## 1. Introduction

Belalai Gajah or Sabah Snake Grass, scientifically known as *Clinacanthus nutans* (Burm.f.) Lindau, is a perennial herb that belongs to the family of Acanthaceae. The herb *C. nutans* is a herb found natively in Thailand, Malaysia, Indonesia, and China [1–3]. Fresh leaves of *C. nutans* are used by traditional Thai healers to treat inflammation [4] and viral infection [5–7]. Previous studies also demonstrated that the *C. nutans* leave extract possesses antioxidant [8, 9], anticancer [9–11], antimicrobial [9], analgesic [12], antidiabetic [13], immunomodulatory [14, 15], and wound healing properties, [16] which are all attributed to the phytochemicals it contains. *C. nutans* plant was reported to contain steroids, terpenoids [17], phenolics [18–20], and other bioactive substances such as sulfur-containing glucosides [21], chlorophyll derivatives [22, 23], benzenoids [10], and lipids [24]. These compounds are known to have bioactivities that are beneficial to human health. However, postharvest processing can influence the level of phenolic constituents and their bioactivity in plants.

Myocardial infarction remains a major cause of heart failure and contributes to high morbidity and mortality worldwide. Despite the availability of advanced therapeutics, it is still challenging to reverse heart remodeling caused by cardiomyocyte loss [25]. Studies have reported that the adult heart possesses endogenous cardiac c-kit cells that can regenerate dead myocardium [26, 27], and they can be activated from the quiescent state by growth factors [28] or by factors and matrices secreted by mesenchymal stem cells [29–31]. However, growth factors are expensive and limited by challenges such as low plasma stability, short biological half-lives, and low specificity to target organs [32]. An alternative remedy sourced from the plant has been studied in *Geum japonicum* extract and was reported to be helpful for myocardial regeneration [33]. Thus, plant-derived compounds could be another therapeutic regimen for cardiac repair. *C. nutans*, a widely grown local herb, may also prove to be helpful for treating heart disease. The herb can potentially prevent insulin resistance-induced cardiovascular diseases by reducing metabolic effects and transcriptional changes induced by a high fat and high cholesterol diet [34].

Plant metabolomic analysis involves the application of mathematical and statistical calculations of metabolite variations in plants of different species under different processing conditions [35]. Sample variation analyses are commonly performed with analytical tools such as Fourier transform infrared (FTIR) spectroscopy, liquid chromatography-mass spectroscopy (LCMS), and nuclear magnetic resonance (NMR). Multivariate data analysis (MVDA), including principal component analysis (PCA) and partial least squares to latent structures (PLS), helps reduce the dimensionality of big datasets to facilitate visualization of sample distribution and to correlate the chemical profile with the tested activity.

Studies have shown that extraction parameters may be the main factors in determining the polyphenol content and the bioactivity of constituents of plant extracts [36]. Therefore, the goal of this study was to optimize postharvest processing of *C. nutans* leaves (i.e., extraction parameters) by identifying the best extraction solvent, solid-liquid ratio, duration of extraction, and the number of extraction cycles. We employed attenuated total reflectance-FTIR (ATR-FTIR) spectroscopy to profile the metabolites present in the samples and chemometrics techniques to investigate the quality and safety of *C. nutans* extracts after postharvest processing. We also tested the effects of these extracts on the viability of mouse endogenous cardiac c-kit cells.

## 2. Materials and Methods

### 2.1. Chemicals and Reagents

Water was purified using a Milli-Q water purifier system (Millipore, Milford, MA, USA), and analytical grade ethanol (99.7%) was purchased from R&M (Semenyih, Selangor, Malaysia). Penicillin streptomycin (PenStrep), Dulbecco's Modified Eagle Medium: Nutrient Mixture F-12 (DMEM/F12), Dulbecco's Phosphate-Buffered Saline (DPBS), TrypLE Express Enzyme, and presto blue were all obtained from Gibco (Waltham, MA, USA). Folin-Ciocalteu (FC) reagent, gallic acid, and sodium carbonate were purchased from Sigma (St. Louis, MO, USA).

### 2.2. Plant Material

Dried *C. nutans* leaves were purchased from Herbagus Sdn Bhd (Bertam, Pulau Pinang, Malaysia). A plant specimen was stored at the Herbarium Unit, School of Biological Sciences, Universiti Sains Malaysia, Penang, Malaysia (voucher no. SK 1980/11). The dried leaves were pulverized into a fine powder using a grinder (Ultra Centrifugal Mill ZM200, Retsch, Haan, Germany). The powder was then sealed in a plastic bag and kept at room temperature until further use.

### 2.3. Extraction

The chemometric experimental design-based optimization techniques were adapted from [37–39] with modifications to determine the conditions that resulted in the highest bioactivities from the PLS model in chemometrics analysis. Briefly, four operating parameters were tested, including the following parameters: (1) solvent polarity (aqueous, 25%, 50%, 75%, and 100% ethanol), (2) solid-liquid ratio (1 : 5, 1 : 10, 1 : 15, 1 : 20, and 1 : 25), (3) ultrasonic-assisted extraction duration (10, 20, 30, 40, and 50 min), and (4) number of extraction cycles (1, 2, 3, 4, and 5 cycles). Dried *C. nutans* leaves were powdered using a herb grinder (Retsch, ZM 200, Haan, Germany) and mixed with solvent at their respective ratio. Experiments for parameter 1 were carried out in three cycles at 1 : 15 solid-liquid ratio and 30 min of ultrasonic duration. The subsequent parameter 2 study used the optimized solvent polarity from parameter 1, set at the same ultrasonic duration and number of extraction cycles. Meanwhile, parameter 3 experiments used an optimized solvent polarity and the solid-liquid ratio. Parameter 4's experiments took place using the optimized conditions from the previous experiments. The mixtures went through a sonication process according to their respective conditions. Extracts were centrifuged at 6000 rpm for 15 min. Supernatants collected were vacuum filtered through Whatman No.1 filter paper (Little Chalfont, Bucks, UK) and lyophilized under centrifugal vacuum using a laboratory evaporator (Genevac EZ-2 Series, Ipswich, England). The lyophilized extracts were kept in a desiccator before analysis.

### 2.4. ATR-FTIR Analysis

FTIR spectra were obtained using a Thermo Nicolet iS10 FTIR spectrometer (Madison, WI, USA), equipped with a smart iTR accessory. A small amount of each extract (*n* = 3 technical replicates) was applied neatly on the ATR crystal and scanned in absorption mode. IR measurements were made at the mid-IR range between 4000 and 650 cm^−1^, with a spectral resolution of 4 cm^−1^.

### 2.5. Spectral Processing

All spectra were converted into percentage transmittance form for spectrum preprocessing. Each IR spectrum was then subjected to baseline correction and smoothing using Omnic software (Version 7.3, Madison, WI, USA). Data exported from Omnic software were compiled into an MS Excel (Version 2016, Redmond, WA, USA) file and subjected to SIMCA-P software (Version 13.0, Umetrics, Umeå, Sweden) for MVDA analysis [40].

### 2.6. Total Phenolic Content

The total phenolic content (TPC) of each extract was assessed by the FC method, as described previously [41, 42] with some modifications. Gallic acid was used as the standard for this assay. For each 10 *µ*L test sample or “standard,” 600 *µ*L of FC reagent (10-fold diluted) and 400 *µ*L of sodium carbonate solution were added, mixed well, and kept in the dark for 60 min. Next, 150 *µ*L of each reaction mixture was transferred to a 96-well plate. The absorbance value was acquired at 765 nm using a FLUOstar® Omega microplate reader (BMG Labtech, Ortenberg, Germany). A gallic acid calibration curve was constructed and used to calculate the TPC of each sample. Results were expressed as mg gallic acid equivalent (GAE)/g dry weight (dw) of extract.

### 2.7. Cytotoxicity Assay

Cardiac c-kit cells (CCs) were isolated from C57/BL mice, as reported previously. Passage 5 CCs were seeded into a 96-well plate with 10% FBS at a density of 1000 cells per well and incubated at 37°C and 5% CO_2_. Next, 5 mg of each *C. nutans* extract was prepared with 2.5 mL of serum-free DMEM/F12 to yield a stock concentration of 2 mg/mL. The extracts went through a sonication process to ensure solubility and were filtered across a 0.22 *µ*m syringe filter. Extracts then were serially diluted to 50, 100, 500, and 1000 *µ*g/mL. After one hour of incubation, *C. nutans* crude leave extracts were added to CCs and incubated for 96 h. All experiments were conducted in triplicate. Serum-free DMEM/F12 was served as the control of the study. Cytotoxicity of *C. nutans* was examined using the presto blue assay. Fluorescence readings were taken using the FLUOstar® Omega microplate reader with fluorescence excitation and emission wavelengths of 540–570 nm and 580–610 nm, respectively. Cell viability in percentage was calculated using the following formula:

(1) $$\begin{array}{c} \%\text{ Viability}=\frac{\text{Fluorescence reading of}\left(\text{Test Sample}-\text{Background}\right)}{\text{Fluorescence reading of}\left(\text{Control}-\text{Background}\right)}\times 100\%. \end{array}$$

### 2.8. Statistical Analysis

Data obtained from TPC and CCs cytotoxicity assays were processed in MS Excel and analyzed using SPSS (Version 22.0, IBM, Armonk, NY, USA). One-way analysis of variance (ANOVA) was used to examine for significant differences. Unit variance (UV) scaling was performed as the pretreatment step in MVDA via the SIMCA-P software. PCA was used as the initial pattern recognition technique for visualization of the clustering of samples in score scatter plots. Correlation of samples and bioactivities was observed in the biplot of PLS. Hotelling's T2 was among the diagnostic tools used to identify strong outliers. *R*^2^ (goodness of fit) and *Q*_2_ (goodness of prediction) estimates were used to validate the PLS model. The predictive ability of the PLS model was also tested using cross-validated analysis of variance (CV-ANOVA) and the 100 random permutation function. Variable importance in projection (VIP) values ≥1 served as the cut-off point for the identification of the top influential signals for the PLS model [43].

## 3. Results and Discussion

### 3.1. Effects of Extraction Parameters on TPC

The parameters indicated a range of extraction yields for crude *C. nutans* extracts, as shown in Table S1. The TPC of crude extracts (Table 1) was impacted profoundly by solvent polarity, S-L ratio, extraction duration, and the number of extraction cycles. The 25% ethanol crude extract had the highest TPC (45.99 mg GAE/g dw), and the value was statistically significantly higher (*p* < 0.05) than that of all other solvent polarities.

Other studies reported a similar result [44, 45] in which *C. nutans* extract produced by reflux extraction yielded the highest TPC at the same solvent polarity (25% ethanol). This higher TPC yield was because the higher water content in the 25% ethanol system caused the plant material to swell, increasing the contact between solvent and plant matrix [46]. Although polyphenols are mostly polar, a highly polar solvent system, such as water, commonly produces extracts with a high level of impurities such as sugars, soluble proteins, and organic acids and can hinder the extraction of phenolic compounds [47–49]. In the current study, higher ethanol concentrations (50–100% ethanol) decreased TPC value, and absolute ethanol (100%) reduced the phenolic yield in the extract.

The results were expressed in mean ± standard error of the mean (SEM), where *n* = 3. Values marked with the same letter subscript represent comparison within the same extraction parameter, while values with the same superscripts portrayed significant differences (*p* < 0.05) within samples of the same parameter set. Statistical significance was analyzed using SPSS one-way ANOVA.

Based on these results, the 25% ethanol solvent system was suitable for extracting the optimal number of polyphenols. All samples differed significantly in TPC value (*p* < 0.05), except for the 50% and 75% ethanol extracts. Therefore, when testing for different S-L ratios in the subsequent experiments, 25% ethanol was used.

When 25% ethanol extracts, with the five different S-L ratios, were tested, the sample extracted at 1 : 15 S-L ratio had the highest TPC value (47.58 mg GAE/g dw). The TPC value increased as the S-L ratio increased to 1 : 15. However, from that point, the value started to decrease. This change may be due to the force driven by the concentration gradient between solid and solvent, as described by principles of mass transfer. A higher S-L ratio increases the concentration gradient and diffusion rate to allow greater extraction of solids (phenolic compounds) by solvent until equilibrium is reached [22]. The TPC value of the 1 : 15 S-L ratio sample was significantly greater (*p* < 0.05) than that of the other S-L ratio samples tested, except for the 1 : 10 S-L ratio sample. The selection of the 1 : 15 S-L ratio for subsequent testing was due to its higher TPC value.

When 25% ethanol extracts, with an S-L ratio of 1 : 15, were tested through three extraction cycles, the TPC of the highest value (58.52 mg GAE/g dw) came from the 30 min duration extract. The TPC value increased significantly with increasing duration of extraction (*p* < 0.05) up to 30 min but declined thereafter. In addition to the overheating effect from extended extraction time, long-term exposure to light and oxygen in the extraction medium could cause phenolic oxidation [50]. Additionally, Fick's second law of diffusion could be at work in longer durations (i.e., solute concentrations in the plant matrix and solvent system could reach final equilibrium [51]). These results suggest that prolonged extraction duration does not increase the yield of phenolic compounds.

Extraction usually is repeated several times, and extracts are then combined to enhance the extraction efficiency [52, 53]. When testing the 25% ethanol extract with an S-L ratio of 1 : 15 and 30-minute extraction duration with different numbers of extraction cycles, multiple cycles of extraction were found to improve the extraction yield of polyphenols. Three extraction cycles produced the highest TPC value (45.49 mg GAE/g dw), which was significantly greater than that of the cycle numbers (*p* < 0.01). In another study, three extraction cycles showed greater yields of TPC and TFC than two and four extraction cycles [54]. Hence, three extraction cycles were optimal for extracting polyphenol compounds from *C. nutans* leaves.

### 3.2. Effects of Extraction Parameters on CC Activity

**Figure 1:**
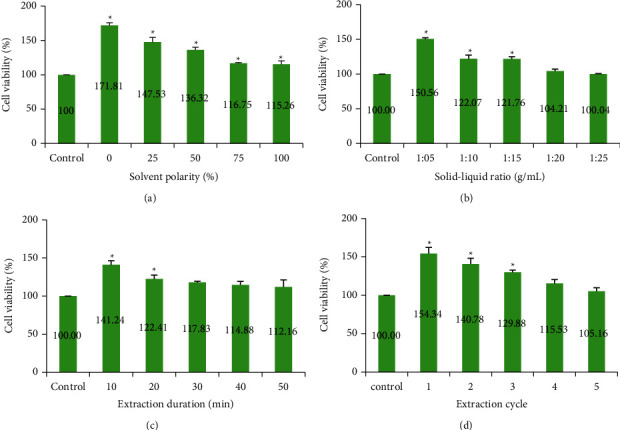
(a–d) Comparison of cell viability of CSCs after 96 hr treatment with *C. nutans* extracts against control (DMEM/F12) at different extraction parameters 1–4 (solvent polarity, solid-liquid ratio, extraction duration, and the cycle of extraction). Statistical analysis was conducted using Dunnett's test. ^*∗*^indicates that samples were significantly different from control (*p* < 0.05). Results were expressed in mean ± standard error of the mean (SEM), where *n* = 3.

CCs have the potential in salvaging the damaged heart's manipulation of the cells in vitro and boost its function in repairing damaged myocardium and can serve as an integrative strategy in increasing the therapeutic effects and outcome. Solvent polarity, S-L ratio, extraction duration, and the number of extraction cycles had profound impacts on the viability of CCs (Table 1). To examine the cytotoxicity of the *C. nutans* leave extracts, CCs were treated with extracts for 96 h (Figures 1(a)–1(d)). *C. nutans* was not cytotoxic to CCs. Instead, it promoted cell growth, even at its highest concentration (1000 *µ*g/mL). Cells treated with the *C. nutans* aqueous extract (0% ethanol) at 100 *µ*g/mL had significantly greater viability than cells treated with the other concentrations (*p* < 0.05). However, CC viability decreased with increasing ethanol concentrations, demonstrating a dose-dependent relationship (Figure 1(a)). The suggestion is that highly polar polyphenols in the aqueous extract were more favorable to the survival and growth of CCs. As the S-L ratio increased, the viability of CCs declined (Figure 1(b)). CCs treated with the 1 : 5 S-L ratio treatment exhibited the highest viability—significantly greater than that of the control (*p* < 0.05). This indicates that polyphenols, extracted at lower S-L ratios and lower ethanol concentrations, were more favorable to CC growth.

Moreover, CC viability decreased with increasing extraction time and the number of extractions cycles (Figures 1(c) and 1(d)). The highest CC viability was observed with *C. nutans* extract single extraction in 10 min. These observations collectively suggest that extracts with the highest TPC do not necessarily contribute to the highest cell viability. Nonetheless, a shorter exposure time to the ethanol-containing extraction solvent results in less solvent contact with the leaves could also contribute to better CC viability. It appears that not only increased extraction cycles produce extracts with more compounds or impurities that could affect CC viability [55], the exposure time to the extraction solvent could also affect CC bioactivity.

### 3.3. FTIR Spectrum Analysis

*C. nutans* leave extracts, scanned through ATR cells, had their spectra compared within their respective parameters to assess the functional group differences and similarities among the extracts. Based on the comparison among parameter 1 (solvent polarity) tested extracts, several peaks were similar for all samples. These include peaks assigned to O–H stretching (3500–3200 cm^–1^), asymmetric stretching of C-H (2935–2915 cm^–1^), C=C aromatic stretch (∼1600 cm^−1^), O-H bending of phenol or tertiary alcohol groups (1410–1310 cm^–1^), primary alcohol groups (∼1050 cm^–1^), C-H bending of end methylene (∼900 cm^–1^), and C-H bending of benzene rings (∼820 and ∼770 cm^–1^). A profound difference was observed between the aqueous and ethanol *C. nutans* extracts (Table 2), with 10 and 11 known peaks, respectively. Symmetrical C-H stretching was absent in aqueous extract. Ester (*υ* C=O) peaked at 1726 cm^−1^ and differentiated the absolute ethanol extract from all other parameter 1 tested samples. Furthermore, the presence of C-O stretching from ester or glycoside near 1246 cm^−1^ made the absolute ethanol extract more distinctive than the others. IR analysis indicated the presence of aromatic compounds in all extracts attributed to the aromatic benzene ring stretching (C=C) at ∼1600 cm^−1^ and C-H bending (∼820 cm^−1^ and ∼770 cm^–1^). Nitro groups (asymmetrical N-O stretching) of aromatics at ∼1514 cm^−1^ were observed in both aqueous and aqueous-ethanolic extracts. Meanwhile, symmetrical N-O stretching of the aromatic nitro groups (1360–1290 cm^–1^), visible in the IR peak of the aqueous-ethanolic extracts (25–75%), became a prerequisite for identifying aqueous-ethanolic *C. nutans* extracts. Functional groups such as hydroxyl, carbonyl, and aromatic rings indicated in *C. nutans* extracts, characterized the presence of phenolic and flavonoids compounds.

In parameter 2 tests (solid-liquid ratio [S-L ratio] variation), there were six major peaks identified for all samples (Table S2). They showed a possible association with asymmetric stretching of C-H, indicating the presence of methylene at 2935–2915 cm^−1^. Aromatic compounds were revealed in all of the *C. nutans* extracts ascribed by the presence of C=C stretching (2935–2915 cm^–1^), asymmetrical N-O stretching from nitro groups (1550–1475 cm^–1^), and C-H bending of the benzene ring (∼820 cm^–1^). The vibrations of bending O-H (1410–1310 cm^–1^) and stretching C-O (∼1050 cm^–1^) further confirmed the presence of phenol and alcoholic groups in all samples. Extracts with S-L ratios of 1 : 10, 1 : 15, and 1 : 20 exhibited almost an identical fingerprint. Meanwhile, extract with the S-L ratio of 1 : 5 was defined by the absence of symmetrical N–O stretching in the nitro group (1360–1290 cm^–1^) and the benzene ring C-H bending near the region at 770 cm^−1^. C-H stretching of alkene (3091 cm^–1^) was observed in the 1 : 25 S-L ratio extracts. However, secondary alcohol groups (∼1070 cm^–1^) and end methylene (∼900 cm^–1^) were not found in the 1 : 25 S-L ratio extract.

Extracts produced from different extraction times (parameter 3) did not vary significantly. Twelve peaks were assigned into their respective functional groups (Table S3). There was no difference in chemical profiles found among the samples extracted from shorter periods (10, 20, and 30 min). However, extracts with a longer extraction time (50 min) lacked secondary alcohol groups (*υ* C-O) at ∼1070 cm^−1^. This lack of secondary alcohol groups was probably due to the effect of overheating by sonication [56], signifying that prolonged sonication could potentially cause the decomposition of bioactive constituents.

The number of extraction cycles (parameter 4) had slight effects on the FTIR fingerprint of the samples (Table S4). Extracts extracted at one and two-cycle(s) lacked benzene ring (*δ* C-H) at ∼770 cm^−1^, which might be due to short sonication exposure. Bending C-H of the benzene ring (∼770 cm^–1^) was found in extracts where the number of extraction cycles increased to 3, 4, and 5. However, long-term exposure to ultrasonic cavitation can cause certain compounds to degrade. For instance, crude extract acquired from five extraction cycles eliminated O-H stretching (3500–3200 cm^–1^) of hydroxyl and C-O stretching (∼1070 cm^–1^) from secondary alcohol groups. 3-cycled extraction was shown as the optimal condition to attain the most functional compound groups in *C. nutans* extract. A peak of C=O ester stretching at 1202 cm^−1^ was only found in the 3-cycled sonication extract, suggesting that the number of extraction cycles can alter the extract's quality.

### 3.4. Classifications of Samples of Each Parameter Set by PCA

**Figure 2:**
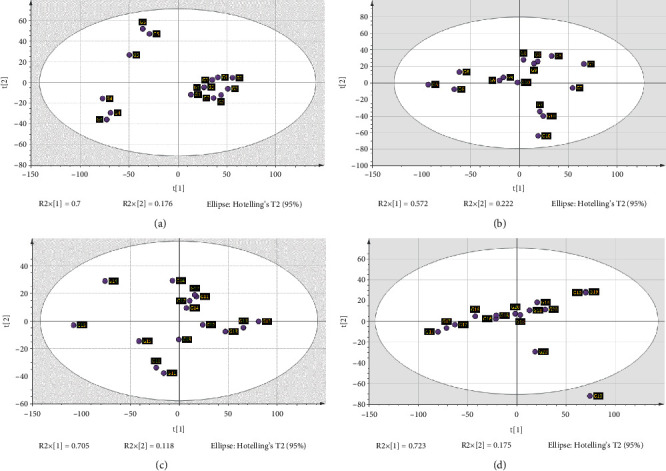
(a–d) PCA-X score scatter plots of parameters 1–4 (solvent polarity, solid-liquid ratio, extraction duration, and the cycle of extraction) at PC1 and PC2.

PCA was conducted using the processed ATR-FTIR data to illustrate the pattern of distribution of samples within each parameter set (Figures 2(a)–2(d)). The first two principal components in the first parameter set yielded a total variation of 87.6%, followed by the second parameter with 79.4% of the total variation. PCA for the third and fourth parameters achieved maximum variation of 82.3% and 89.8%, respectively. Based on PCA, all tested groups within each parameter set exhibited an acceptable value of goodness of fit (*R*^2^*X* [cum] = 0.876, 0.794, 0.822, and 0.898, respectively) and predictability (*Q*^2^ [cum] = 0.826, 0.681, 0.662, and 0.709, respectively). The difference between *R*^2^*X* (cum) and *Q*^2^ (cum) was within 0.2, confirming that the PCA models for each parameter set were reliable.

For the first parameter set (solvent polarity) in the score scatter plot, PC1 separated G1 (aqueous), G2 (25% ethanol), and G3 samples (50% ethanol) from G4 (75% ethanol) and G5 (100% ethanol) samples (Figure 2(a)). An assessment was made of Hotelling's T2 values to identify the presence of any strong outliers at two levels, specifically at 95% and 99% confidence intervals. Among the tested samples, none were identified as outliers as they all fell within the interval of 99%. It was deducible that based on the polarity of polyphenols, PC1 made a differentiation because G1 to G3 samples may possess a similar majority of highly polarised polyphenols. The close clustering within G1 to G3 group samples was not distinctively different from the unsupervised PCA model. However, the G4 and G5 samples discriminated from the rest of the groups.

For the second parameter set (S-L ratio), only G6 samples (1 : 5 S-L ratio) were distinctively different from the other group samples based on PC1. G2, G7, G8, and G9 samples (1 : 15, 1 : 10, 1 : 20, and 1 : 25, respectively) overlapped themselves in the PCA plot, which shows that these samples shared similar compounds to a significant degree. Hotelling's T2 showed no strong outliers present in these groups.

For samples from the third parameter set (extraction times), G2 (30 min extraction) was the only well-clustered group. PC1 divided the samples into two major parts with G10 (10 min extraction), G12 (40 min extraction), and G13 (50 min extraction) in the positive region and G11 (20 min extraction) and G13 in the negative region of the plot. One sample from G10 and G13 overlapped with G12 samples. These could be described as moderate outliers, which may have arisen from technical error. Meanwhile, no strong outliers were observed.

Groups from the number of extraction cycles (parameter 4) were closely clustered with each other. G17 (5 extraction cycles) samples tended to overlap with G2 (3 extraction cycles) samples, and this could be a moderate outlier. A single strong outlier was detected in G16 (4 extraction cycles) samples, as it fell outside the 95% Hotelling's T2 ellipse, but the datum was accepted in the analysis because the confidence interval was within 99%.

### 3.5. Correlations of Sample Profile with Bioactivities via PLS

**Figure 3:**
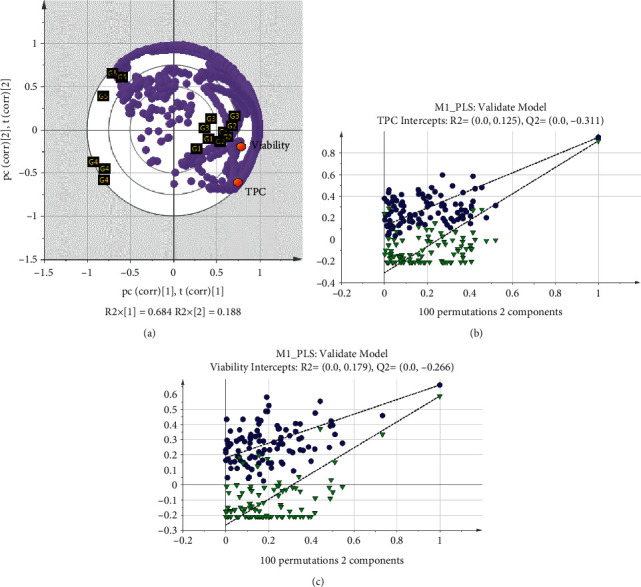
(a) PLS biplot showed the distribution of samples from different solvent polarity and their relationship to both TPC and CSCs activities at PC1 and PC2. (b, c) Permutation plot of *R*^2^ and *Q*^2^ for TPC and CSCs viability, respectively.

All tested parameter sets were subjected to PLS analysis. However, only the first parameter set (solvent polarity) passed the internal *R*^2^*Y* and *Q*^2^ cumulative cross-validation stage in SIMCA-P software. Validation of the PLS model for solvent polarity occurred, with acceptable goodness of fit (*R*^2^*Y* = 0.801) and good predictive power (*Q*^2^ cumulative = 0.741). The biplot of the PLS model revealed the distribution of samples and their correlation to the TPC and CCs activities. Data showed that G1 and G2 clustered closely for both of the activities (Figure 3(a)). Considering the previous results, significantly high TPC values in the G1 and G2 samples made them suitable for CCs growth with high viability. VIP analysis, carried out to identify the X-variables that had the most influence on the PLS model, identified a total of 1786 signals with VIP ≥1. The first tenth of VIP values ≥1 was selected as the top influential signals contributing to the PLS models at PC1 and PC2. In addition to the evaluation of VIP, CV-ANOVA was employed to confirm the significance of the prediction model in the PLS model [57]. CV-ANOVA showed that the correlation between the chemical profile and TPC and CCs activity was significant (*p* < 0.05). The indication was that the solvent polarity used in extraction could significantly affect the chemical profile and bioactivities. The PLS biplot was affirmed with 100 random permutations to assess the reliability of the PLS model [57]. Based on the result, the *Q*^2^ regression line that is colored green was negatively intercepted (Figures 3(b) and 3(c)). Furthermore, *R*^2^ and *Q*^2^ plots were well intercepted to each other in activities, particularly in TPC, and were in line with a previous study by Chew et al. [43]. Therefore, the effect of solvent polarity on bioactivities presented in the PLS model is reliable for the study.

## 4. Conclusion

The application of the ATR-FTIR chemometrics approach revealed sample variation in each tested parameter set via their respective PCA models. PLS model demonstrated the relationship among samples from the first parameter set and their correlation to TPC and cytotoxicity results. From the chemometric-based optimization techniques, solvent polarity was the most influential factor affecting the observed chemical differences and bioactivities of *C. nutans* extracts. G1 (aqueous, S-L ratio of 1 : 15, and three cycles of extraction) and G2 (25% ethanol, S-L ratio of 1 : 15, and three cycles of extraction) were identified as the optimum conditions for extracting *C. nutans* extracts with the ideal TPC values and no toxicity to CCs. MVDA results were subjected to several validation tools such as *R*^2^ and *Q*^2^, VIP, CV-ANOVA, and permutation plots to confirm the reliability of the PLS model. These approaches proved effective as a guide to optimize postharvest processing parameters and provide extracts with the greatest TPC bioactivities and CC viability.

## Figures and Tables

**Table 1:** Effect of extraction parameters on TPC and CSCs viability.

Ethanol concentration (%)	TPC (mg/g) at 5000 *µ*g/mL	Viability (%) at 100 *µ*g/mL
0	44.66 ± 0.83_a_^a,b,c,d^	171.81 ± 4.06_a_^a,b,c,d^
25	45.99 ± 0.29_a_^a,e,f,h^	147.53 ± 6.80_a_^a,e,f^
50	34.01 ± 0.50_a_^b,e,i^	136.32 ± 3.77_a_^b,g^
75	31.19 ± 0.44_a_^c,f,j^	116.75 ± 1.03_a_^c,e,g^
100	11.40 ± 0.88_a_^d,h,i,j^	115.26 ± 4.93_a_^d,f^

Solid-liquid ratio (g/mL)	TPC (mg/g) at 5000 *µ*g/mL	Viability (%) at 100 *µ*g/mL
1 : 05	40.58 ± 1.15_b_^a,b^	150.56 ± 1.67_b_^a,b,c,d^
1 : 10	44.54 ± 1.75_b_^c,d^	122.07 ± 5.21_b_^a,e,f^
1 : 15	47.58 ± 0.40_b_^a,e,f^	121.76 ± 3.21_b_^b,g,h^
1 : 20	36.33 ± 1.06_b_^c,e^	104.21 ± 2.87_b_^c,e,g^
1 : 25	35.26 ± 0.30_b_^b,d,f^	100.04 ± 0.52_b_^d,f,h^

Ultrasonic duration (min)	TPC (mg/g) at 5000 *µ*g/mL	Viability (%) at 100 *µ*g/mL
10	41.17 ± 0.39_c_^a,b,c,d^	141.24 ± 5.12_c_^a,b,c^
20	49.11 ± 1.62_c_^a,e,f,g^	122.41 ± 5.11
30	58.52 ± 0.74_c_^b,e,h^	117.83 ± 1.67_c_^*a*^
40	56.46 ± 0.04_c_^c,f,j^	114.88 ± 4.45_c_^*b*^
50	36.61 ± 0.88_c_^d,g,h,i^	112.16 ± 9.08_c_^*c*^

Extraction cycle	TPC (mg/g) at 5000 *µ*g/mL	Viability (%) at 100 *µ*g/mL
1	23.37 ± 0.83_d_^a,b,c,d^	154.34 ± 7.98_d_^a,b^
2	35.01 ± 0.86_d_^a,e^	140.78 ± 7.36_d_^*c*^
3	45.49 ± 0.29_d_^b,e,f,g^	129.88 ± 2.91
4	36.18 ± 0.93_d_^c,f^	115.53 ± 5.00_d_^*a*^
5	33.35 ± 0.93_d_^d,g^	105.16 ± 4.72_d_^b,c^

**Table 2:** List of functional group differences reported in *C. nutans* aqueous and ethanol extracts along with their tentative identification from parameter 1.

Test sample wavenumber (cm^−1^)	Reference wavenumber (cm^−1^)	Functional group assignment	Tentative identification
*Aqueous extract*			
3229	3570–3200	*υ* O-H, H-bonded, hydroxy group	Polyhydroxy compound
2928	2935–2915	*υ* as CH_2_	Lipids, protein
1604	1615–1580	*υ* C=C-C, aromatic ring	Aromatic compound
1514	1555–1485	*υ* NO_2_, nitrogen-oxy group	Aromatic nitro compound
1402	1410–1310	*δ* O-H, alcoholic group	Phenol or tertiary alcohol
1045	∼1050	*υ* C-O, alcoholic group	Primary alcohol
934, 903, 871	995–850	*υ* P-O-C	Aromatic phosphates
836	840–815	Nitrate ions	Nitrate compound
777	800–700	*υ* C-Cl	Aliphatic chloro compound
651	660–630	*υ* CH_3_-S	Thioethers

*Absolute ethanol extract*			
3273	3570–3200	*υ* O-H, H-bonded, hydroxy group	Polyhydroxy compound
2921	2935–2915	*υ* asymmetric CH_2_	Lipids, protein
2852	2865–2845	*υ* symmetric CH_2_	Lipids, protein
1726	1740–1725	*υ* C=O, carbonyl group	Aldehyde compound
1625	1680–1620	*υ* C=C	Alkenyl
1403, 1370	1410–1310	*δ* O-H, alcoholic group	Phenol or tertiary alcohol
1246	1270–1230	*υф*-O-H, aryl-O stretch	Aromatic ethers
1050	∼1050	*υ* C-O, alcoholic group	Primary alcohol
932, 902, 851	995–850	*υ* P-O-C	Aromatic phosphates
837	840–815	Nitrate ions	Nitrate compound
777	800–700	*υ* C-Cl	Aliphatic chloro compound

^*∗*^note: *υ* = stretching; *δ* = bending.

## Data Availability

All the data generated or analyzed in this study are included in this manuscript. The ATR-FTIR data for parameters 2, 3, and 4 used to support the findings of this study are included within the supplementary information file.

## Supplementary Materials

Supplementary Materials Table S1: extraction yield (% *w*/*w*) of all the parameters. Table S2: list of functional groups reported in different solid-liquid (S-L) ratios of *C. nutans* extracts along with their tentative identification (parameter 2). Table S3: major similarity peaks of functional groups reported in the duration of *C. nutans* extraction times along with their tentative identification (parameter 3). Table S4: major similarity peaks of functional groups reported in the extraction cycles of *C. nutans* along with their tentative identification (parameter 4).

## References

[1] Chiu H. I., Che Mood C. N. A., Mohamad Zain N. N. (2021). Biogenic silver nanoparticles of *Clinacanthus nutans* as antioxidant with antimicrobial and cytotoxic effects. *Bioinorganic Chemistry and Applications*.

[2] Devasvaran K., Baharom N. H., Chong H. W. (2020). Quality assessment of *Clinacanthus nutans* leaf extracts by GC-MS-based metabolomics. *Current Science*.

[3] Mat Yusuf S. N. A., Che Mood C. N. A., Ahmad N. H., Sandai D., Lee C. K., Lim V. (2020). Optimization of biogenic synthesis of silver nanoparticles from flavonoid-rich *Clinacanthus nutans* leaf and stem aqueous extracts. *Royal Society Open Science*.

[4] Yoosook C., Panpisutchai Y., Chaichana S., Santisuk T., Reutrakul V. (1999). Evaluation of anti-HSV-2 activities of *Barleria lupulina* and *Clinacanthus nutans*. *Journal of Ethnopharmacology*.

[5] Charuwichitratana S., Wongrattanapasson N., Timpatanapong P., Bunjob M. (1996). Herpes zoster: treatment with *Clinacanthus nutans* cream. *International Journal of Dermatology*.

[6] Janwitayanuchit W., Suwanborirux K., Patarapanich C., Pummangura S., Lipipun V., Vilaivan T. (2003). Synthesis and anti-herpes simplex viral activity of monoglycosyl diglycerides. *Phytochemistry*.

[7] Wanikiat P., Panthong A., Sujayanon P., Yoosook C., Rossi A. G., Reutrakul V. (2008). The anti-inflammatory effects and the inhibition of neutrophil responsiveness by *Barleria lupulina* and *Clinacanthus nutans* extracts. *Journal of Ethnopharmacology*.

[8] Pannangpetch P., Laupattarakasem P., Kukongviriyapan V., Kukongviriyapan U., Kongyingyoes B., Aromdee C. (2007). Antioxidant activity and protective effect against oxidative hemolysis of *Clinacanthus nutans* (burm f) lindau. *Songklanakarin Journal of Science and Technology*.

[9] Arullappan S., Rajamanickam P., Thevar N., Kodimani C. (2014). In vitro screening of cytotoxic, antimicrobial and antioxidant activities of *Clinacanthus nutans* (acanthaceae) leaf extracts. *Tropical Journal of Pharmaceutical Research*.

[10] Yong Y. K., Jun J. T., Soek S. T. (2013). *Clinacanthus nutans* extracts are antioxidant with antiproliferative effect on cultured human cancer cell lines. *Evidence-Based Complementary and Alternative Medicine*.

[11] Yakop F., Aisyah Abd Ghafar S., Keong Yong Y. (2018). Silver nanoparticles *Clinacanthus nutans* leaves extract induced apoptosis towards oral squamous cell carcinoma cell lines. *Artificial Cells, Nanomedicine, and Biotechnology*.

[12] Satayavivad J., Bunyaoraphatsara N., Kitisiripornkul S., Tanasomwang W. (1996). Analgesic and anti-inflammatory activities of extract of *Clinacanthus nutans* lindau. *Journal of Pharmaceutical Sciences*.

[13] Wong F.-C., Yong A.-L., Peir-Shan Ting E., Khoo S.-C., Ong H.-C., Cha T.-T. (2014). Antioxidant, metal chelating, anti-glucosidase activities and phytochemical analysis of selected tropical medicinal plants. *Iranian Journal of Pharmaceutical Research*.

[14] Sriwanthana B., Chavalittumrong P., Chompuk L. (1996). Effect of *Clinacanthus nutans* on human cell-mediated immune response in vitro. *Thai Journal of Pharmaceutical Sciences*.

[15] Lau K. W., Lee S. K., Chin J. H. (2014). Effect of the methanol leaves extract of *Clinacanthus nutans* on the activity of acetylcholinesterase in male mice. *Journal of Acute Disease*.

[16] Kongkaew C., Chaiyakunapruk N. (2011). Efficacy of *Clinacanthus nutans* extracts in patients with herpes infection: systematic review and meta-analysis of randomised clinical trials. *Complementary Therapies in Medicine*.

[17] Dampawan P., Huntrakul C., Reutrakul V., Raston C. L., White A. H. (1977). Constituents of *Clinacanthus nutans* and the crystal structure of LUP-20(29)-ene-3-one. *Journal of the Science Society of Thailand*.

[18] Phua Q. Y., Subramaniam S., Lim V., Chew B. L. (2018). The establishment of cell suspension culture of sabah snake grass (*Clinacanthus nutans* (burm.F.) lindau). *In Vitro Cellular & Developmental Biology—Plant*.

[19] Lin C. M., Chen H. H., Lung C. W., Chen H. J. (2021). Recent advancement in anticancer activity of *Clinacanthus nutans* (burm. F.) lindau. *Evidence-Based Complementary and Alternative Medicine*.

[20] Chelyn J. L., Hasyima Omar M., Syaidatul Akmal Mohd Yousof N., Ranggasamy R., Isa Wasiman M., Ismail Za. (2014). Analysis of flavone C -glycosides in the leaves of *Clinacanthus nutans* (burm. F.) lindau by HPTLC and HPLC-UV/DAD. *The Scientific World Journal*.

[21] Teshima K.-I., Kaneko T., Ohtani K. (1998). Sulfur-containing glucosides from *Clinacanthus nutans*. *Phytochemistry*.

[22] Sakdarat S., Shuyprom A., Pientong C., Ekalaksananan T., Thongchai S. (2009). Bioactive constituents from the leaves of *Clinacanthus nutans* lindau. *Bioorganic & Medicinal Chemistry*.

[23] Sittiso S., Ekalaksananan T., Pientong C., Sakdarat S., Charoensri N., Kongyingyoes B. (2010). Effects of compounds from *Clinacanthus nutans* on dengue virus type 2 infection. *Srinagarind Medical Journal*.

[24] Tuntiwachwuttikul P., Pootaeng-On Y., Phansa P., Taylor W. C. (2004). Cerebrosides and a monoacylmonogalactosylglycerol from *Clinacanthus nutans*. *Chemical and Pharmaceutical Bulletin*.

[25] Alam M. A., Ishfaq M. F., Khanam B. (2016). Is cardiac stem cell therapy a new horizon of heart regeneration: literature review. *Molecular Biology*.

[26] Laflamme M. A., Murry C. E. (2011). Heart regeneration. *Nature*.

[27] Assmus B., Zeiher A. M. (2013). Early cardiac retention of administered stem cells determines clinical efficacy of cell therapy in patients with dilated cardiomyopathy. *Circulation Research*.

[28] Nadal-Ginard B., Ellison G. M., Torella D. (2014). The cardiac stem cell compartment is indispensable for myocardial cell homeostasis, repair and regeneration in the adult. *Stem Cell Research*.

[29] Leong Y. Y., Ng W. H., Umar Fuaad M. Z. (2019). Mesenchymal stem cells facilitate cardiac differentiation in Sox2 ‐expressing cardiac C‐kit cells in coculture. *Journal of Cellular Biochemistry*.

[30] Ng W. H., Ramasamy R., Yong Y. K. (2019). Extracellular matrix from decellularized mesenchymal stem cells improves cardiac gene expressions and oxidative resistance in cardiac C-kit cells. *Regenerative Therapy*.

[31] Ng W. H., Umar Fuaad M. Z., Azmi S. M. (2019). Guided evaluation and standardisation of mesenchymal stem cell culture conditions to generate conditioned medium favourable to cardiac c-kit cell growth. *Cell and Tissue Research*.

[32] Rebouças J. D. S., Santos-Magalhães N. S., Formiga F. R. (2016). Cardiac regeneration using growth factors: advances and challenges. *Arquivos Brasileiros de Cardiologia*.

[33] Li M., Yu C. M., Cheng L. (2006). Repair of infarcted myocardium by an extract of *Geum japonicum* with dual effects on angiogenesis and myogenesis. *Clinical Chemistry*.

[34] Sarega N., Imam M. U., Esa N. M., Zawawi N., Ismail M. (2016). Effects of phenolic-rich extracts of *Clinacanthus nutans* on high fat and high cholesterol diet-induced insulin resistance. *BMC Complementary and Alternative Medicine*.

[35] Kim H. K., Choi Y. H., Verpoorte R. (2011). NMR-based plant metabolomics: where do we stand, where do we go?. *Trends in Biotechnology*.

[36] Yuan Y., Liu Y., Liu M. (2017). Optimization extraction and bioactivities of polysaccharide from wild *Russula griseocarnosa*. *Saudi Pharmaceutical Journal*.

[37] Guerrero Peña A., Anguebes Franseschi F., Castelán Estrada M. (2014). Fourier transform infrared-attenuated total reflectance (ftir-atr) spectroscopy and chemometric techniques for the determination of adulteration in petrodiesel/biodiesel blends. *Química Nova*.

[38] Nasruddin N. I. N., Jamil M. S. M., Zakaria I., Zubairi S. I. (2018). Optimization of noodle formulation using commercialized empty fruit bunch palm oil carboxylmethyl cellulose (CMC) and flours with different protein content. *Jurnal Teknologi*.

[39] Abd-ElSalam H.-A. H., Gamal M., Naguib I. A., Al-Ghobashy M. A., Zaazaa H. E., Abdelkawy M. (2021). Development of green and efficient extraction methods of quercetin from red onion scales wastes using factorial design for method optimization: a comparative study. *Separations*.

[40] Lim V., Gorji S. G., Daygon V. D., Fitzgerald M. (2020). Untargeted and targeted metabolomic profiling of Australian indigenous fruits. *Metabolites*.

[41] Singleton V. L., Rossi J. A. (1965). Colorimetry of total phenolics with phosphomolybdic-phosphotungstic acid reagents. *American Journal of Enology and Viticulture*.

[42] Kähkönen M. P., Hopia A. I., Vuorela H. J. (1999). Antioxidant activity of plant extracts containing phenolic compounds. *Journal of Agricultural and Food Chemistry*.

[43] Chew H. W., Chong H. W., Rezaei K., Chew B. L., Lim V. (2018). Chemometric profiling of *Clinacanthus nutans* leaves possessing antioxidant activities using. *Chiang Mai Journal of Science*.

[44] Che Sulaiman I. S., Basri M., Fard Masoumi H. R. (2017). Effects of temperature, time, and solvent ratio on the extraction of phenolic compounds and the anti-radical activity of *Clinacanthus nutans* lindau leaves by response surface methodology. *Chemistry Central Journal*.

[45] Sultana B., Anwar F., Przybylski R. (2007). Antioxidant activity of phenolic components present in barks of *Azadirachta indica*, *Terminalia arjuna*, *Acacia nilotica*, and *Eugenia jambolana* lam. trees. *Food Chemistry*.

[46] Hemwimol S., Pavasant P., Shotipruk A. (2006). Ultrasound-assisted extraction of anthraquinones from roots of *Morinda citrifolia*. *Ultrasonics Sonochemistry*.

[47] Cacace J. E., Mazza G. (2003). Mass transfer process during extraction of phenolic compounds from milled berries. *Journal of Food Engineering*.

[48] Herodež S., Hadolin M., Škerget M., Knez Ž. (2003). Solvent extraction study of antioxidants from Balm (*Melissa officinalis* L.) leaves. *Food Chemistry*.

[49] Goldsmith C., Vuong Q., Stathopoulos C., Roach P., Scarlett C. (2014). Optimization of the aqueous extraction of phenolic compounds from olive leaves. *Antioxidants*.

[50] Chan S. W., Lee C. Y., Yap C. F., Aida W. M. W., Ho C. W. (2009). Optimisation of extraction conditions for phenolic compounds from limau purut (*Citrus hystrix*) peels. *International Food Research Journal*.

[51] Salar R. K., Purewal S. S., Bhatti M. S. (2016). Optimization of extraction conditions and enhancement of phenolic content and antioxidant activity of pearl millet fermented with *Aspergillus awamori* MTCC-548. *Resource-Efficient Technologies*.

[52] Chen X.-X., Wu X.-B., Chai W.-M. (2013). Optimization of extraction of phenolics from leaves of *Ficus virens*. *Journal of Zhejiang University—Science B*.

[53] Galanakis C. M. (2018). *Polyphenols: Properties, Recovery, and Applications*.

[54] Milugo T. K., Omosa L. K., Ochanda J. O. (2013). Antagonistic effect of alkaloids and saponins on bioactivity in the quinine tree (*Rauvolfia caffra* sond.): further evidence to support biotechnology in traditional medicinal plants. *BMC Complementary and Alternative Medicine*.

[55] Eriksson L., Trygg J., Wold S. (2008). CV-ANOVA for significance testing of PLS and OPLS® models. *Journal of Chemometrics*.

[56] Rafiee Z., Jafari S. M., Alami M., Khomeiri M. (2011). Microwave-assisted extraction of phenolic compounds from olive leaves; a comparison with maceration. *Journal of Animal and Plant Sciences*.

[57] Ke C., Li A., Hou Y. (2016). Metabolic phenotyping for monitoring ovarian cancer patients. *Scientific Reports*.

