# Supplementary material for: Vibrational Spectroscopy-Based Chemometrics Analysis of Clinacanthus nutans Extracts after Postharvest Processing and Extract Effects on Cardiac C-Kit Cells
Source: Evid Based Complement Alternat Med. 2022 Feb 23;2022:1967593. doi: 10.1155/2022/1967593 (PMC8890836; doi:10.1155/2022/1967593)
Supplement: Supplementary Materials — Table S1: extraction yield (% w/w) of all the parameters. Table S2: list of functional groups reported in different solid-liquid (S-L) ratios of C. nutans extracts along with their tentative identification (parameter 2). Table S3: major similarity peaks of functional groups reported in the duration of C. nutans extraction times along with their tentative identification (parameter 3). Table S4: major similarity peaks of functional groups reported in the extraction cycles of C. nutans along with their tentative identification (parameter 4). [file 1967593.f1.docx]

Table S1: Extraction yield (% w/w) of all the parameters.

| **Ethanol Concentration (%)** | **Extraction yield (%w/w)** |
| --- | --- |
| 0 | 12.83±0.31 |
| 25 | 11.13±0.25 |
| 50 | 9.27±0.55 |
| 75 | 7.93±0.15 |
| 100 | 8.03±0.21 |
| **Solid-liquid ratio (g/mL)** | **Extraction yield (%w/w)** |
| 1:05 | 11.87±0.06 |
| 1:10 | 11.53±0.35 |
| 1:15 | 10.93±0.15 |
| 1:20 | 9.07±0.15 |
| 1:25 | 9.23±0.06 |
| **Ultrasonic duration (min)** | **Extraction yield (%w/w)** |
| 10 | 11.47±0.38 |
| 20 | 10.97±0.12 |
| 30 | 9.77±0.15 |
| 40 | 9.07±0.15 |
| 50 | 8.87±0.06 |
| **Extraction cycle** | **Extraction yield (%w/w)** |
| 1 | 12.20±0.17 |
| 2 | 11.63±0.21 |
| 3 | 10.27±0.15 |
| 4 | 8.73±0.21 |
| 5 | 9.07±0.15 |

The results were expressed in mean ± standard deviation (SD), where n = 3.

Table S2: List of functional groups reported in different solid-liquid (S-L) ratios of *C. nutans* extracts along with their tentative identification (parameter-2).

| **Test sample wavenumber (cm^-1^)** | **Reference wavenumber (cm^-1^)** | **Functional group assignment** | **Tentative identification** |
| --- | --- | --- | --- |
| **S-L ratio=1:5** | | | |
| 3259 | 3570-3200 | 𝜐 O-H, H-bonded, Hydroxy group | Polyhydroxy compound |
| 2926 | 2935-2915 | 𝜐 Asymmetric CH_2_ | Lipids, protein |
| 1605 | 1615-1580 | 𝜐 C=C-C, Aromatic ring | Aromatic compound |
| 1514 | 1555-1485 | 𝜐 NO2, Nitrogen-oxy group | Aromatic nitro compound |
| 1402 | 1410-1310 | 𝛿 O-H, Alcoholic group | Phenol or tertiary alcohol |
| 1202 | 1210-1150 | 𝜐 C-N | Tertiary amine |
| 1075 | 1150-1000 | 𝜐 C-F | Aliphatic fluoro compound |
| 1046 | ~1050 | 𝜐 C-O, Alcoholic group | Primary alcohol |
| 934, 904 | 995-850 | 𝜐 P-O-C | Aromatic phosphate |
| 835 | 840-815 | Nitrate ions | Nitrate compound |
| 654 | 660-630 | 𝜐 CH_3_-S | Thioethers |
| **S-L ratio=1:10** | | | |
| 3140 | 3570-3200 | 𝜐 O-H, H-bonded, Hydroxy group | Polyhydroxy compound |
| 2928 | 2935-2915 | 𝜐 Asymmetric CH_2_ | Lipids, protein |
| 1598 | 1615-1580 | 𝜐 C=C-C, Aromatic ring | Aromatic compound |
| 1512 | 1555-1485 | 𝜐 NO_2_, Nitrogen-oxy group | Aromatic nitro compound |
| 1396 | 1410-1310 | 𝛿 O-H, Alcoholic group | Phenol or tertiary alcohol |
| 1289 | 1340-1250/1350-1280 | 𝜐 C-N | Aromatic primary/secondary amine |
| 1078 | 1150-1000 | 𝜐 C-F | Aliphatic fluoro compound |
| 1045 | ~1050 | 𝜐 C-O, Alcoholic group | Primary alcohol |
| 901 | 995-850 | 𝜐 P-O-C | Aromatic phosphate |
| 839 | 840-815 | Nitrate ions | Nitrate compound |
| 779 | 800-700 | 𝜐 C-Cl | Aliphatic chloro compound |
| 655 | 660-630 | 𝜐 CH_3_-S | Thioethers |
| **S-L ratio=1:15** | | | |
| 3231 | 3570-3200 | 𝜐 O-H, H-bonded, Hydroxy group | Polyhydroxy compound |
| 2927 | 2935-2915 | 𝜐 Asymmetric CH_2_ | Lipids, protein |
| 1596 | 1615-1580 | 𝜐 C=C-C, Aromatic ring | Aromatic compound |
| 1513 | 1555-1485 | 𝜐 NO_2_, Nitrogen-oxy group | Aromatic nitro compound |
| 1396 | 1410-1310 | 𝛿 O-H, Alcoholic group | Phenol or tertiary alcohol |
| 1291 | 1340-1250/1350-1280 | 𝜐 C-N | Aromatic primary/secondary amine |
| 1079 | 1150-1000 | 𝜐 C-F | Aliphatic fluoro compound |
| 1045 | ~1050 | 𝜐 C-O, Alcoholic group | Primary alcohol |
| 935, 883 | 995-850 | 𝜐 P-O-C | Aromatic phosphate |
| 837 | 840-815 | Nitrate ions | Nitrate compound |
| 776 | 800-700 | 𝜐 C-Cl | Aliphatic chloro compound |
| 649 | 660-630 | 𝜐 CH_3_-S | Thioethers |
| **S-L ratio=1:20** | | | |
| 2927 | 2935-2915 | 𝜐 Asymmetric CH_2_ | Lipids, protein |
| 1595 | 1615-1580 | 𝜐 C=C-C, Aromatic ring | Aromatic compound |
| 1512 | 1555-1485 | 𝜐 NO2, Nitrogen-oxy group | Aromatic nitro compound |
| 1395 | 1410-1310 | 𝛿 O-H, Alcoholic group | Phenol or tertiary alcohol |
| 1288 | 1340-1250/1350-1280 | 𝜐 C-N | Aromatic primary/secondary amine |
| 1076 | 1150-1000 | 𝜐 C-F | Aliphatic fluoro compound |
| 1045 | ~1050 | 𝜐 C-O, Alcoholic group | Primary alcohol |
| 902 | 995-850 | 𝜐 P-O-C | Aromatic phosphate |
| 839 | 840-815 | Nitrate ions | Nitrate compound |
| 780 | 800-700 | 𝜐 C-Cl | Aliphatic chloro compound |
| 655 | 660-630 | 𝜐 CH_3_-S | Thioethers |
| **S-L ratio=1:25** | | | |
| 3091 | 3150-3000 | 𝜐 Unsaturation C=C-H/ aromatic rings | Vinyldene C-H stretch |
| 2926 | 2935-2915 | 𝜐 Asymmetric CH_2_ | Lipids, protein |
| 1603 | 1615-1580 | 𝜐 C=C-C, Aromatic ring | Aromatic compound |
| 1514 | 1555-1485 | 𝜐 NO_2_, Nitrogen-oxy group | Aromatic nitro compound |
| 1402 | 1410-1310 | 𝛿 O-H, Alcoholic group | Phenol or tertiary alcohol |
| 1291 | 1340-1250/1350-1280 | 𝜐 C-N | Aromatic primary/secondary amine |
| 1200 | 1210-1150 | 𝜐 C-N | Tertiary amine |
| 1045 | ~1050 | 𝜐 C-O, Alcoholic group | Primary alcohol |
| 934 | 995-850 | 𝜐 P-O-C | Aromatic phosphate |
| 838 | 840-815 | Nitrate ions | Nitrate compound |
| 776 | 800-700 | 𝜐 C-Cl | Aliphatic chloro compound |
| 655 | 660-630 | 𝜐 CH_3_-S | Thioethers |

Table S3: Major similarity peaks of functional groups reported in the duration of *C. nutans* extraction times along with their tentative identification (parameter-3).

| **Test sample wavenumber (cm^-1^)** | **Reference wavenumber (cm^-1^)** | **Functional group assignment** | **Tentative identification** |
| --- | --- | --- | --- |
| 3221, 3206,3231, 3240, 3265 | 3570-3200 | 𝜐 O-H, H-bonded, Hydroxy group | Polyhydroxy compound |
| 2928, 2926, 2927, 2925, 2926 | 2935-2915 | 𝜐 Asymmetric CH_2_ | Lipids, protein |
| 1600, 1599, 1596, 1599, 1605 | 1615-1580 | 𝜐 C=C-C, Aromatic ring | Aromatic compound |
| 1513 | 1555-1485 | 𝜐 NO_2_, Nitrogen-oxy group | Aromatic nitro compound |
| 1396, 1395, 1396, 1396, 1402 | 1410-1310 | 𝛿 O-H, Alcoholic group | Phenol or tertiary alcohol |
| 1288, 1281, 1291, 1287 | 1340-1250/1350-1280 | 𝜐 C-N | Aromatic primary/secondary amine |
| 1176, 1174, 1202 | 1210-1150 | 𝜐 C-N | Tertiary amine |
| 1210-1150 | 𝜐 C-N | 𝜐 C-F | Aliphatic fluoro compound |
| 1200-1100 | ~1050 | 𝜐 C-O, Alcoholic group | Primary alcohol |
| 935, 896, 936, 897, 935, 883, 898, 935, 904 | 995-850 | 𝜐 P-O-C | Aromatic phosphate |
| 837, 838 | 840-815 | Nitrate ions | Nitrate compound |
| 778, 776, 777, 776 | 800-700 | 𝜐 C-Cl | Aliphatic chloro compound |
| 655, 649 | 660-630 | 𝜐 CH_3_-S | Thioethers |

Table S4: Major similarity peaks of functional groups reported in the extraction cycles of *C. nutans* along with their tentative identification (parameter-4).

| **Test sample wavenumber (cm^-1^)** | **Reference wavenumber (cm^-1^)** | **Functional group assignment** | **Tentative identification** |
| --- | --- | --- | --- |
| 3275, 3250, 3231 | 3570-3200 | 𝜐 O-H, H-bonded, Hydroxy group | Polyhydroxy compound |
| 2927, 2925 | 2935-2915 | 𝜐 Asymmetric CH_2_ | Lipids, protein |
| 1601, 1598, 1596, 1591, 1602 | 1615-1580 | 𝜐 C=C-C, Aromatic ring | Aromatic compound |
| 1513 | 1555-1485 | 𝜐 NO_2_, Nitrogen-oxy group | Aromatic nitro compound |
| 1396, 1398, 1402 | 1410-1310 | 𝛿 O-H, Alcoholic group | Phenol or tertiary alcohol |
| 1289, 1291, 1288, 1287 | 1340-1250/1350-1280 | 𝜐 C-N | Aromatic primary/secondary amine |
| 1202 | 1210-1150 | 𝜐 C-N | Tertiary amine |
| 1113 | 1200-1100 | 𝜐 sulfur-oxy group | Sulfonates |
| 1081, 1080, 1079, 1077 | 1150-1000 | 𝜐 C-F | Aliphatic fluoro compound |
| 1045, 1044 | ~1050 | 𝜐 C-O, Alcoholic group | Primary alcohol |
| 936, 935, 937, 897,883, 869, 902 | 995-850 | 𝜐 P-O-C | Aromatic phosphate |
| 838, 837, 839 | 840-815 | Nitrate ions | Nitrate compound |
| 777, 780 | 800-700 | 𝜐 C-Cl | Aliphatic chloro compound |
| 655, 649, 660, 652 | 660-630 | 𝜐 CH_3_-S | Thioethers |
